# Purely Off-Clamp Laparoscopic Partial Nephrectomy Stands the Test of Time: 15 Years Functional and Oncologic Outcomes from a Single Center Experience

**DOI:** 10.3390/curroncol30010092

**Published:** 2023-01-15

**Authors:** Aldo Brassetti, Umberto Anceschi, Alfredo Maria Bove, Francesco Prata, Manuela Costantini, Mariaconsiglia Ferriero, Riccardo Mastroianni, Leonardo Misuraca, Gabriele Tuderti, Giulia Torregiani, Marco Covotta, Michele Gallucci, Giuseppe Simone

**Affiliations:** 1 Department of Urology, IRCCS “Regina Elena” National Cancer Institute, 00144 Rome, Italy; 2 Department of Anesthesiology, IRCCS “Regina Elena” National Cancer Institute, 00144 Rome, Italy

**Keywords:** laparoscopy, long term outcomes, partial nephrectomy, renal cancer, survival

## Abstract

Background: Nephron-sparing surgery represents the gold standard treatment for organ-confined renal tumors. We present 15-years of outcomes after off-clamp laparoscopic partial nephrectomy (ocLPN). Methods: a retrospective analysis was performed on patients who underwent ocLPN between May 2001 and December 2005. Baseline demographic, clinical, pathologic, surgical, functional and survival data were collected. The Kaplan–Meier method evaluated group-specific oncologic outcomes at 5, 10 and 15 years and the log rank test assessed differences between groups. The same analysis investigated the probabilities of developing a significant renal function impairment (sRFI) and achieving *ROMeS*. Cox analyses identified predictors of this latter tricomposite outcome. Results: We included 63 patients whose median tumor size was 3 cm (IQR:2–4). At 15 years, the chances of developing local recurrence, metachronous renal cancers or distant metastases were 2 ± 2%, 23 ± 6% and 17 ± 5%, respectively. Consequently, disease-free, cancer-specific and overall-survival probabilities were 68 ± 6%, 90 ± 4% and 72 ± 6%. MCRSS and UCISS well predicted oncologic outcomes. Overall, nine (14%) patients experienced an sRFI and 33 (52%) achieved *ROMeS*. Age (HR: 1.046; *p* = 0.033) and malignant histology (low-risk cancers HR: 3.233, *p* = 0.048) (intermediate/high risk cancers HR: 5.721, *p* = 0.023) were independent predictors of *ROMeS* non-achievement. Conclusions: At 15 years from ocLPN, most of patients will experience both excellent functional and oncologic outcomes.

## 1. Introduction

Kidney cancer ranks among the most prevalent in the United States [1] and the vast majority are renal cell carcinomas (RCC). Extirpative therapies have always been the mainstay treatment options but, as our understanding of cancer biology has matured, there has been a paradigm shift from radical nephrectomy to nephron-sparing surgery (NSS) and partial nephrectomy (PN) is nowadays considered the gold standard for organ-confined diseases [2]. 

Clamping of the renal hilar vasculature is traditionally performed during PN to minimize bleeding and theoretically enhance surgical accuracy. To reduce the risk for ischemia-reperfusion injury, however, many different strategies were proposed such as off-clamp enucleation, which we pioneered [3].

First proposed by Clayman in the 90s [4], the propagation of the laparoscopic approach was initially hampered by the inherent complexity of the procedure, a steep learning curve and longer operation times (OT). As more experience was accumulated, results of minimally-invasive surgery were proven non-inferior to those of open PN, with the added advantages of reduced intraoperative blood loss, faster recovery and improved cosmesis [4]. 

Regardless, its recent widespread, laparoscopic PN (LPN) has always been a challenging procedure, mainly performed in high-volume referral centers and only few series were published, with a limited follow-up. The aim of the present study is to report functional and oncologic outcomes after 15 years of off-clamp LPN (ocLPN) at our center.

## 2. Materials and Methods

### 2.1. Patients and Dataset

After institutional review board approval, our prospectively maintained database was queried for patients undergone LRP for organ-confined (cT1-2N0M0) renal tumors, between May 2001 and December 2005. 

The following data were collected:

1. Age, gender, race, body mass index (BMI), relevant comorbidities (hypertension and diabetes), ASA score;
2. Tumor side and clinical size;
3. Clamping technique, hemoglobin drop, perioperative complications and length of hospital stay (LOS);
4. Serum creatinine levels before surgery and at follow-up. At each assessment, estimated glomerular filtration rates [eGFR] was calculated by means of the Chronic Kidney Disease Epidemiology Collaboration formula [5] and stratified according to the National Kidney Foundation and the Kidney Disease Outcomes Quality Initiative [6]. Any new onset of Chronic Kidney Disease (CKD) stage ≥ IIIa (or ≥ IV, in patients with a baseline already <60 mL/min/1.73 m^2^) was defined as “significant renal function impairment” (sRFI);
5. Final histology and staging [7]. Patients with clear cell (ccRCC) and non-clear cell renal cell carcinomas (non-ccRCC) were stratified into risk groups according to the Mayo Clinic Risk Stratification System (MCRSS) [8] and the University of California Integrated Staging System (UCISS) [9], respectively;
6. Tumor recurrence time and site. Disease-free (DFS), cancer-specific (CSS) and overall survival (OS).

The above-mentioned data were used to outline a binary variable for the achievement of a tricomposite outcome called *ROMeS*, which combines the absence of recurrence, overall Mortality and eGFR significant deterioration at 15 years [10].

### 2.2. Postoperative Care and Surgical Technique

The follow-up schedule included serum creatinine assessment, whole-body computed tomography and abdominal ultrasonography with concomitant chest X-ray scans, alternately carried out at 6-months intervals [11].

An off-clamp approach was always attempted. The surgical technique has been previously described elsewhere [12,13]. All the surgeries were performed by a single experienced surgeon (M.G.)

### 2.3. Study Objective

Our aim was to report 15 years of functional and oncologic outcomes of ocLPN.

### 2.4. Statistical Analysis

Means and standard deviations were used to report continuous variables; frequencies and proportions for categorical ones. Chi square and Mann–Whitney tests compared categorical and continuous variables, respectively. 

The Kaplan–Meier (KM) method was performed to evaluate group-specific oncologic outcomes, computed at 5, 10 and 15 years after surgery; the log rank test was used to assess statistically significant differences between groups. The same model investigated the probabilities of sRFI onset and *ROMeS* achievement over time. Univariable and multivariable Cox regression models were performed to identify predictors of this latter composite outcome. For all tests, significance level was set at a *p* value of <0.05. Statistical analysis was performed using the Statistical Package for Social Science v. 25.0 (IBM, Somers, NY, USA).

## 3. Results

Overall, 63 consecutive patients were included in the analysis. Most of them were men (60%) with a median age of 64 years (IQR: 54–71) and a BMI of 24.5 (IQR: 21.8–27.8); three (4%) presented with solitary kidneys (Table 1). The median tumor size was 3 cm (IQR: 2–4). 

While 14 patients (22%) were diagnosed with a benign tumor, 40 (63%) and 9 (15%) harbored clear cell and non-clear cell cancers, respectively; 6 (9%) of these malignancies were intermediate/high risk (IR/HR) diseases, according to the Mayo Clinic and University of Southern California stratification systems. The positive surgical margins rate was 5% (*n* = 3). Median LOS was 5 (IQR: 3–6) days (Table 2) and 12 patients overall required blood transfusion (6 in both groups; *p* = 0.65); no severe recurrences requiring surgery or intensive care unit admission were observed (data not shown). All the treated patients underwent a purely off-clamp approach, and no case of intraoperative conversion to radical nephrectomy was observed. 

**Figure 1:**
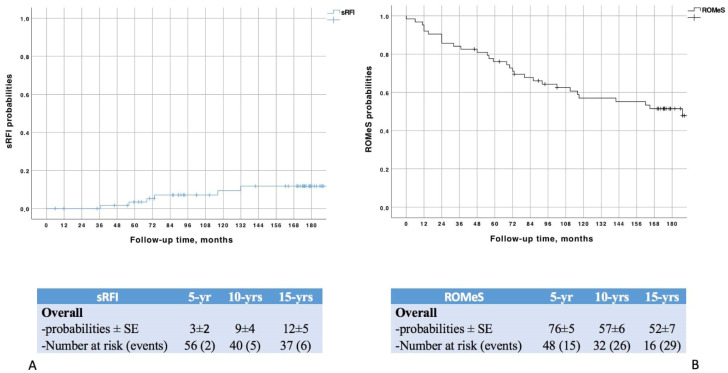
Probabilities of developing a significant renal function impairment (**A**) and (**B**) achieving ROMeS.

The median time of follow-up was 171 months (IQR: 88–187). Within the study period, the sRFI rate was 14% (*n* = 9) (Figure 1); seven (11%) patients were diagnosed with newly onset CKD stage IIIa while two (3%) developed a stage IV.

**Figure 2:**
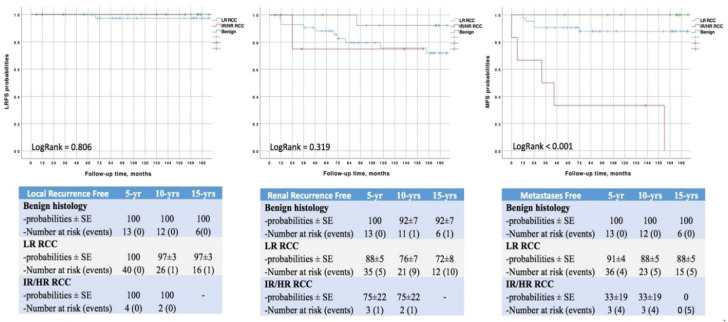
Probabilities of local recurrence, renal recurrence and metastasis, according to MCRSS/UCISS stratification systems.

**Figure 3:**
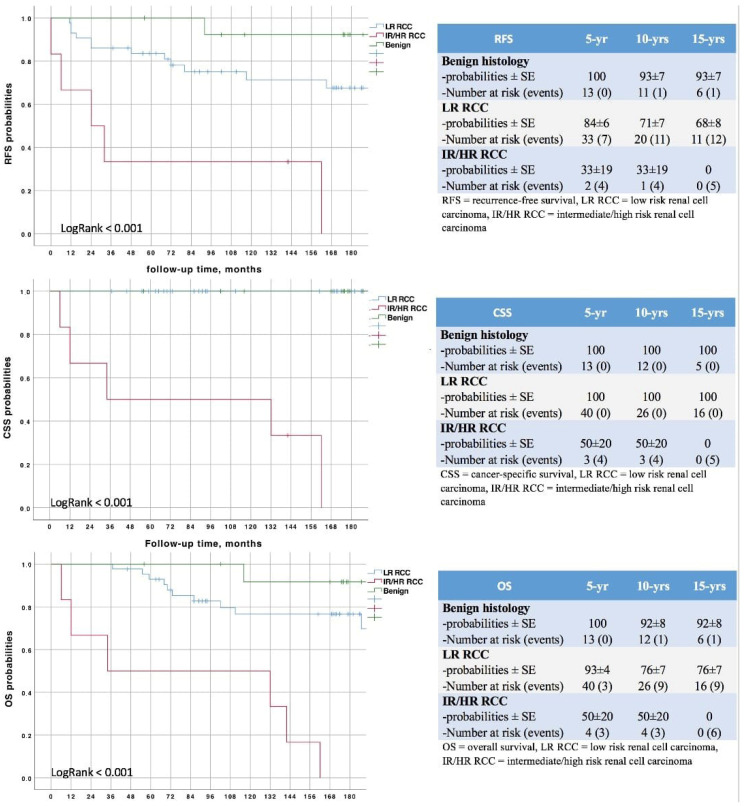
Survivals according to histology and Mayo Clinic and University of Southern California risk stratification systems.

While local recurrences were uncommon (*n* = 1; 1.5%), 12 patients (19%) were diagnosed with metachronous tumors in the ipsi- or contralateral kidney and 10 (16%) developed distant metastases during the study period. The risk of metastatic spread was significantly higher in IR/HR RCC (log rank *p* < 0.001; Figure 2). Fifteen-year DFS, CSS and OS probabilities were 68 ± 6%, 90 ± 4% and 72 ± 6%, respectively, and varied according to histology and MCRSS/UCISS risk groups (all *p* < 0.001) (Figure 3). 

Overall, 52% of patients (*n* = 33) achieved 15-yr ROMeS: these were younger (57 years vs. 71 years; *p* = 0.001), commonly female (52% vs. 23%; *p* = 0.021) and often harbored a benign tumor (33% vs. 10%; *p* = 0.026) (Table 1 and Table 2). At multivariable Cox analysis, age (HR: 1.046; *p* = 0.033) and malignant histology (LR RCC vs. Benign HR: 3.233, *p* = 0.048) (IR/HR RCC vs. Benign HR: 5.721, *p* = 0.023) were independent predictors of ROMeS nonachievement (Table 3).

## 4. Discussion

International guidelines recommend NSS whenever feasible, and mostly to treat cT1 renal tumors. Although percutaneous thermal ablation techniques represent a viable option and should be considered in case of frail patients with small (<3 cm) masses [14,15], PN is nowadays considered the gold standard for organ-confined diseases [2] and, with the introduction of the robotic surgical platforms, it can be also offered to selected patients with large neoplasms [16].

The first series of LPN was published in 1995 [4]. Initially hampered by a significant learning curve and longer OT, the widespread of this minimally-invasive surgical intervention has finally occurred as NSS has been set as the gold standard treatment for renal tumors, “whenever feasible” [17]. Over the last decades, LPN increased, and open surgery was gradually overcome, although the former remained a technically demanding surgical procedure, mainly performed in high-volume referral centers. Furthermore, the advent of robotic systems encouraged the utilization of minimally-invasive approaches in this setting, so that the number of these procedures significantly rose [18]. Between 2008 and 2013 in England, most PNs were performed with a conventional or robot-assisted laparoscopic approach [19]. Indeed, robotics improved ergonomics and dexterity thus facilitating renorrhaphy and shortening the learning curve [20], however the cost of the surgical systems remains a major concern [21] which is only partially covered by savings related with fewer inpatient admissions and outpatient visits [19]. 

Before October 2004, 63 patients underwent off-clamp LPN at our center. The median tumor size was 3 cm (IQR:2–4), in line with most other series from that era [22,23]. Only later, in fact, grounded evidence proved that a laparoscopic approach was feasible and safe also in selected >4 cm renal masses [24]. 

Clamping of the renal hilum is traditionally performed during PN to minimize intraoperative blood loss and enhance visualization of the surgical field. Every minute of warm ischemia may increase the risk of post-operative renal function deterioration [25], although conflicting evidence was reported concerning this topic. Two recent RCTs found no difference in functional outcomes between on- vs. off-clamp approaches [26,27]: most enrolled patients, however, presented with low-nephrometry renal masses and, consequently, short average warm ischemia times were observed in the on-clamp arms. Conversely, we proved that WIT does affect postoperative renal function and this can be easily observed in patients with a solitary kidney (which cannot rely on a contralateral healthy organ to cope with the surgical injury) [28,29,30] and large renal tumors (that usually require >20 min of WITs) [16]. Laparoscopy is usually associated with longer warm ischemia time, compared to open and robotic surgery [31]. With this regard, Lane et al. demonstrated that patients with a solitary kidney undergoing LPN are at higher risk of postoperative dialysis (10% vs. 0.6%; *p* = 0.001) that those treated with an open PN [29]. The off-clamp technique avoids ischemic injury [32] and proved, both in the imperative [33] and in the elective settings [34], to better preserve post-operative renal function. The trade-off, however, is the increased intraoperative bleeding [32] which could jeopardize the positive surgical margins rate. In the present series, this rate (5%) remained in line with that from other large laparoscopic and open series (0.3–7%) [31] and, at 15 years follow-up, seven patients (11%) were diagnosed with newly onset CKD stage IIIa while two (3%) developed a stage IV. Comparable functional results were observed in other PN cohorts, regardless the open, laparoscopic or robotic approach [35].

As said above, LPN has seen widespread use as NSS has become the reference choice for renal tumors. In the largest cohort (*n* = 1541) comparing open and laparoscopic approaches, 10-year OS rates were not statistically different (72% vs. 78%) while the incidence of distant metastases was significantly higher in the latter group (92% vs. 97%, *p* = 0.02) [22]. Mukkamala et al. recently reported excellent oncologic outcomes a decade after minimally-invasive PN, showing 88% DFS and 71% OS rates [36]. A matched-pair analyses comparing laparoscopic and robotic approaches, with a median follow-up of 5 years, demonstrated no significant differences in local recurrence rate (2.5% vs. 1.5%; *p* = 0.657), distant metastases (2.5% vs. 5%; *p* = 0.764) and cancer-related death (1% vs. 1.5%; *p* = 0.779) [35]. To the best of our knowledge, ours is the first study describing outcomes of LPN 15 years after surgery and the only one reporting on and off-clamp series. According to our analysis, DFS, CSS and OS probabilities were 68 ± 6%, 90 ± 4% and 72 ± 6%, respectively (Figure 3). The risks of distant metastases and recurrence at the tumor bed or at the contralateral kidney were 17 ± 5%, 2 ± 2% and 23 ± 6%, respectively (Figure 2). These results appear in line with those cited above. We also confirmed that both MCRSS [8] and UCISS [9] are excellent predictors of survival outcomes and disease recurrence probabilities (Figure 2 and Figure 3).

Within the study period, 52% of patients overall achieved *ROMeS* [10]; age (HR: 1.046; *p* = 0.033) and malignant histology (LR RCC vs. Benign HR: 3.233, *p* = 0.048) (IR/HR RCC vs. Benign HR: 5.721, *p* = 0.023; Table 2) independently predicted the failure in achieving this favorable tricomposite outcome. Indeed, it is not surprising that patients harboring a malignant tumor are at increased risk of disease recurrence and have reduced survival probabilities according to cancer aggressiveness. Similarly, it is well known that renal function physiologically worsens over time, thus increasing the risk for sRFI onset and finally limiting the chances of achieving desirable long-term functional outcomes [7]. 

Current guidelines recommend up to 5 years of follow-up for patients with localized RCC treated by NSS, although recent studies suggest to extend it further; on the other hand, costs related to radiological monitoring are a matter of concern [37]. Based on these findings, young patients who have undergone ocLPN for a benign tumor have the highest probabilities of achieving good long-term functional and oncologic outcomes and may not benefit from a stringent follow-up schedule. These evidences, together with the nomogram already published by our group [10], may also help defining risk-adapted protocols. 

We acknowledge some limitations to the present study. First, a retrospective analysis was performed on prospectively collected data, with the inherent biases associated with such design. Second, the study population was limited and only representative of a single-surgeon, single high-volume center experience, therefore, results herein reported may be difficult to generalize. However, as said above, the exiguity of the cohort is motivated by the initially restricted spread of LPN due to its steep learning curve and long OT which at the beginning confined this technically demanding surgical procedure in referral centers. Another limitation lies in the fact that only a quarter of the enrolled patients presented with a ≥ T1b tumor. Although there is increasing evidence that a minimally invasive PN is a valuable treatment option in selected cases, most of the published series from that era share the same median tumor size.

## 5. Conclusions

To the best of our knowledge, this is the first study describing 15-yr outcomes of LPN and the only one reporting on an off-clamp series. CSS exceeded 90%, three out of four patients were recurrence-free, sRFI was uncommon and almost half of the study population simultaneously achieved these three outcomes (namely *ROMeS*) at the 15-yr follow-up. Although technically demanding, ocLPN stands the test of time and provides excellent long-term oncologic and functional outcomes. 

## Figures and Tables

**Table 1:** Patients’ characteristics at baseline.

LPN	Overall *n* = 63	No ROMeS *n* = 30 (48%)	ROMeS *n* = 33 (52%)	*p*
**Age, yrs**	64(54–71)	71(62–75)	57(50–66)	0.001
**Male gender, *n*(%)**	39(60%)	23(77%)	16(48%)	0.021
**BMI**	24.5(21.8–27.8)	24(21.7–27.6)	24.5(21.8–28.1)	0.445
**Solitary kidney, *n*(%)**	3(4%)	2(7%)	1(3%)	0.498
**Diabetes, *n*(%)**	14(22%)	6(20%)	8(24%)	0.473
**Hypertension, *n*(%)**	33(52%)	16(53%)	17(52%)	0.491
**ASA score ≧ 3, *n*(%)**	9(14%)	4(13%)	5(15%)	0.434
**Clinical Tumor Size, cm**	3(2–4)	3(2–4)	3(2–4)	0.464

Data are reported as Median (IQR). LPN = laparoscopic partial nephrectomy, BMI = body mass index, ASA = American society of anesthesiologists.

**Table 2:** Outcomes of LPN.

LPN	Overall *n* = 63	No ROMeS *n* = 30 (48%)	ROMeS *n* = 33 (52%)	*p*
**LOS, days**	5 (3–6)	5 (4–6)	5 (3–6)	0.873
**Hb drop, g/dL**	2.5 (2.1–2.7)	2.5 (2.1–3)	2.1 (2.1–2.5)	0.124
**PSM, *n* (%)**	3 (5%)	3 (10%)	0 (0%)	0.063
**Histology, *n* (%)**				0.026
**-Benign**	14 (22%)	3 (10%)	11 (33%)	
**-ccRCC**	40 (63%)	23 (77%)	17 (51%)	
**-non-ccRCC**	9 (15%)	4 (13%)	5 (15%)	
**pT ^†^, *n* (%)**				0.997
**-1a**	47 (75%)	22 (74%)	25 (76%)	
**-1b**	12 (19%)	6 (20%)	6 (18%)	
**-2a**	2 (3%)	1 (3%)	1 (3%)	
**-3a**	2 (3%)	1 (3%)	1 (3%)	
**MCRSS ^‡^, *n* (%)**				0.022
**-Low Risk**	35 (56%)	18 (60%)	17 (52%)	
**-Intermediate/High Risk**	5 (8%)	5 (17%)	0 (0%)	
**UCISS ^¥^, *n* (%)**				0.077
**-Low Risk**	8 (13%)	3 (10%)	5 (15%)	
**-Intermediate/High Risk**	1 (1%)	1 (3%)	0 (0%)	
**eGFR, mL/min/1.73 m** **^2^**				
**-at baseline**	80.1 (64.7–89.9)	80 (64.5–87.9)	80.5 (66.4–92.4)	0.573
**-at last follow-up**	75.1 (62.9–84.9)	55.3 (62.9–75)	79.3 (74.2–95.3)	<0.001
**sRFI, *n* (%)**	9 (14%)	9 (30%)	0 (0%)	<0.001

Data are reported as Median (IQR). ^†^ data are calculated on malignant tumors. ^‡^ data are calculated on clear cell carcinomas. ^¥^ data are calculated on non-clear cell carcinomas. LPN = laparoscopic partial nephrectomy, LOS = length of stay, Hb = hemoglobin, PSM = positive surgical margins, MCRRG = Mayo Clinic Risk Stratification System, UCISS = University of California Los Angeles (UCLA) Integrated Staging System, eGFR = estimated glomerular filtration rate, sRFI = significant renal function impairment.

**Table 3:** Cox regression analyses to identify predictors of *ROMeS* nonachievement.

	Univariable Analysis				Multivariable Analysis			
	HR	95% CI		*p*	HR	95% CI		*p*
		Lower	Higher			Lower	Higher	
**Age**	**1.06**	**1.02**	**1.10**	**0.002**	**1.05**	**1.004**	**1.09**	**0.03**
**Male Gender**	2.80	1.19	6.59	0.02	1.61	0.68	3.82	0.28
**BMI**	0.96	0.87	1.06	0.43	-	-	-	-
**Solitary Kidney**	2.45	0.58	10.34	0.22	-	-	-	-
**Diabetes Mellitus**	0.96	0.77	1.17	0.82	-	-	-	-
**Hypertension**	1.10	0.89	1.23	0.78	-	-	-	-
**ASA score ≧ 3**	0.81	0.88	1.09	0.61	-	-	-	-
**Clinical tumor size**	1.08	0.87	1.35	0.48	-	-	-	-
**Preoperative eGFR**	0.99	0.97	1.02	0.79	-	-	-	-
**LR RCC vs. Benign** **IR/HR RCC vs. Benign**	**3.06** **11.32**	**1.01** **2.79**	**10.26** **45.85**	**0.047** **0.001**	**3.24** **5.72**	**1.01** **1.27**	**10.88** **25.77**	**0.048** **0.023**

BMI = body mass index, eGFR = estimated glomerular filtration rate, LR RCC = low-risk renal cell carcinoma, IR/HR RCC = intermediate/high risk renal cell carcinoma.

## Data Availability

https://gbox.garr.it/garrbox/index.php/s/9Y08C2rEMQy8V8d, accessed on 27 December 2022.
